# Oral health in Africa: a neglected public health priority

**DOI:** 10.3389/froh.2026.1754189

**Published:** 2026-04-08

**Authors:** Myles Nwabunka, Akinyimika O. Sowunmi, Uyi E. Osadolor, Abimbola D. Akinyosoye, Lawrence Nnyanzi, Davies Adeloye, Vida Zohoori

**Affiliations:** 1 Faculty of Humanities, Social and Management Sciences, Amadeus University, Amizi, Nigeria; 2 Faculty of Allied Health Sciences, Amadeus University, Amizi, Nigeria, 3Centre for Population Health and Healthcare, School of Health and Life Sciences, Teesside University, Middlesbrough, United Kingdom; 3 Centre for Public Health, School of Health and Life Sciences, Teesside University, Middlesbrough, United Kingdom; 4 School of Social Sciences, Covenant University, Ota, Nigeria; 5 Department of Biological Sciences, Covenant University, Ota, Nigeria

**Keywords:** Africa, challenges, oral disease burden, oral health, public health

## Abstract

Oral health in Africa is a significantly neglected public health priority, impacting approximately 480 million people (43.7% of the WHO African region population). Despite its critical link to general well-being and other Non-Communicable Diseases (NCDs), the continent faces a high burden of conditions like untreated dental caries, severe periodontal disease, and life-threatening conditions such as Noma. Key challenges include a historic lack of prioritisation in health policy and resource allocation, a critically under-resourced oral health workforce (ratio of 0.44 professionals per 10,000 people), and limited access to care, with high rates of symptomatic visits for tooth removal. Furthermore, there is a significant disparity in research output compared to other continents, reflecting a 10/90 gap in oral health research and a profound lack of longitudinal and clinical trials. Addressing this high burden and low prioritisation demands urgent, evidence-based policy shifts, increased financial investment, strengthening of the workforce, and robust, African-led research to meet global commitments like the WHA 2022 resolution and SDG 3.

## Definition and clinical significance of oral health

Oral health, as defined by the World Health Organisation (WHO), is not merely the absence of disease, but a state that enables individuals to eat, speak, smile and interact socially without pain, discomfort, or embarrassment (1). It includes being free from diseases and disorders such as oral and facial pain, oral cancers, oral infections and sores, periodontal (gum) disease, dental caries (tooth decay), tooth loss and other conditions that impact oral function and psychosocial well-being (2, 3). The World Dental Federation further expands this definition, describing oral health as encompassing the ability to speak, smile, smell, taste, touch, chew, swallow, and convey a range of emotions through facial expressions with confidence and without pain or disease (4). In essence, oral health is a key indicator of general health and quality of life. It is intricately linked with other non-communicable diseases (NCDs), sharing common risk factors such as tobacco use, harmful alcohol consumption and unhealthy diets. Moreover, poor oral health can exacerbate systemic conditions like cardiovascular disease and diabetes, making its neglect a significant public health oversight (5). Oral diseases, particularly dental caries and periodontal disease, are chronic, progressive and cumulative. If untreated, they can result in severe pain, infection, tooth loss and functional impairment. In Africa, these diseases remain poorly addressed, with prevention and early treatment inaccessible primarily to the majority of the population (6).

## Oral health status and challenges in Africa

Despite its critical role in overall well-being, oral health remains a significantly neglected aspect of public health in many African countries. The continent faces a high burden of oral diseases, such as untreated dental caries, severe periodontal disease, and oral cancers (7–9). According to the WHO African region, approximately 43.7% of the population, equating to 480 million people, live with poor oral health (10, 11). Historically, oral health has not been prioritised in health policy agendas, largely due to the continent's overwhelming disease burden from communicable diseases like HIV/AIDS, tuberculosis and malaria (12, 13). As a result, oral health receives minimal attention in resource allocation and health planning. This lack of prioritisation is reflected in health funding allocations, with oral healthcare receiving a fraction of national health budgets in many countries, and access remains limited and uneven (6). Moreover, the oral health workforce across Africa is significantly under-resourced. Dentist-to-population ratios across countries were just 0.44 per 10,000 population, ranging from as low as 0.003 in South Sudan to about 4.29 in Seychelles, while the availability of dental assistants and technicians varies considerably across regions (14). Only 57% of African countries have an oral health policy in place, and in most cases, these are often poorly implemented or underfunded (6). Compounding these structural challenges are socioeconomic inequalities, cultural barriers and lack of integration into primary health care. Populations in rural and underserved areas are particularly vulnerable, with higher rates of untreated oral conditions and poorer access to preventive or restorative care (15).

## Epidemiology of oral conditions in Africa

Dental caries remains the most prevalent oral condition across children and adults. Recent estimates indicate that 28.5% of individuals aged 5 years and older in the WHO African Region have untreated dental caries in their permanent teeth, while 38.6% of children aged 1 to 9 years suffer from untreated caries in their primary teeth (16). In East Africa, studies found that oral disease prevalence ranged from 15.8% to 83%, with a pooled dental caries prevalence of 45.7% and an average decayed, missing, filled teeth (DMFT) score of 1.941 for permanent teeth (17). Among children, pooled data from East Africa also reported an average DMFT score of 2.24, indicating a notable burden of untreated dental caries in primary dentition (18). In Uganda, a separate study among 3–5-year-old children found an early childhood caries (ECC) prevalence of 48.6% with a mean DMFT of 2.04 (19). In South Africa, a study of adults reported that 68% had high DMFT scores, and among older adults, the average DMFT reached 32 years, with tooth loss being the dominant component (20). Among African migrants in Europe, particularly adolescents, the prevalence of dental caries was 89.4%, highlighting the long-term impacts of poor access to oral care in early life (21). In West Africa, the prevalence of oral disorders among adolescents aged 15–19 was over 2.7 million cases in 2019, with Nigeria accounting for the majority (22, 23).

Robust incidence data are limited due to weak oral health surveillance systems across many African countries. However, some conditions, like Noma (cancrum oris), provide an insight into the incidence of severe oral disease. Noma affects 20 per 100,000 children aged 3–6 annually in sub-Saharan Africa, with many cases going undiagnosed or unreported (15, 24). Incidence rates for oral cancers and periodontal disease are also increasing, especially in populations with co-morbidities such as HIV/AIDS, where oral lesions are common (25). In Rwanda, national surveys found that cases of dental caries and gingivitis are rising, particularly among school-age children (17). In Ghana and Nigeria, the frequency of adolescent dental caries is as high as 80%, significantly higher than in many high-income countries (16).

While most oral diseases are non-fatal, some, particularly oral cancers and Noma, carry a significant mortality burden. Oral cancer mortality rates in Africa range from 0.4 to 6.6 per 100,000 people, with poor diagnostic infrastructure contributing to late-stage presentation and poor outcomes (26). For Noma, mortality is estimated at 90% in untreated cases, making it one of the most devastating but neglected oral conditions on the continent (27). Mortality from oral diseases is further complicated by the fact that many patients present only in emergencies, bypassing the window for preventive or early treatment (27, 28). Furthermore, the stigma and disfigurement from oral cancers or Noma often led to psychological trauma, social exclusion and in some cases, suicide (27). Epidemiological patterns of oral diseases in Africa demonstrate wide variability by region, with caries, Noma, and oral cancers contributing significantly to the burden. Table 1 presents a summary of key indicators by region, population, and disease type alongside global comparisons.

**Table 1:** Epidemiological patterns of oral diseases in Africa vs. Global comparison .

Region/Population	Condition	Prevalence	Global comparison
WHO African Region	Untreated dental caries (permanent teeth, age ≥5)	28.5%	35%
WHO African Region	Untreated dental caries (primary teeth, age 1–9)	38.6%	43%
East Africa (children)	DMFT (permanent teeth)	2.24	1.6–3
Uganda (children, age 3–5)	Early Childhood Caries (ECC)	48.6%	23–40%
South Africa (older adults)	Avg. DMFT	32 (tooth loss dominant)	16–26 (depending on country)
West Africa (adolescents)	Oral disorders (age 15–19)	2.7 million	3.5 billion
Sub-Saharan Africa	Noma (age 3–6)	20 per 100,000 children/year	Extremely rare in high-income countries
Africa (oral cancers)	Mortality rate	0.4–6.6 per 100,000	2.5–3.5 per 100,000
Ghana & Nigeria (adolescents)	Dental caries frequency	Up to 80%	20–40%

## Exploring current research, policy, and practice

There is a paucity of studies on oral health in Africa, including leading oral health journals both globally and in Africa. A database search of the Africa-based QI journal on June 24, 2025, highlighted zero Q1 Africa-based journals. A further search of the online database of leading Oral health journals, such as BMC Oral Health publications, over the past 5 years, on June 24, 2025, by combining key oral health keywords with the keyword Africa, yielded 173 research articles on oral health in Africa. Four of these studies were clinical trials, all of which were conducted in Northern Africa. Oral cavity and Oral hygiene have been well covered, as mentioned in 82 and 49 papers, respectively; oral health knowledge or Oral hygiene knowledge was mentioned in 35 papers, periodontal disease in 31 papers, and oral cancer in 10 papers. African-based research on Oral health systems totalled 23 articles, while research articles published on oral health policy totalled 25 papers. A further journal search on topics bordering on “oral health disorders” in Africa yielded 51 results. The results yielded reflect a gap in research into oral health challenges within the African continent. A quick search on clinicaltrials.gov as of June 25, 2025, confirmed that there are currently no clinical trials or longitudinal studies being carried out within African countries and LMIC to harmonise comprehensive data collection. This paucity of research highlights the neglect of oral health within the continent and the lack of sense of urgency about oral health problems among many Africans and their governments.

A further comprehensive search on PubMed for research outputs over the last 25 years in different continents using the Boolean operators (Oral Health) AND (Disease OR Lesion) yielded the results shown below in Table 2.

**Table 2:** Comparison of publication outputs before and after the COVID-19 pandemic (2000–2025).

Region/Database	Search query	Results (2000–2025)	Approx annual output pre-COVID	Publications post-COVID (2021)	Approx. annual output post-COVID
Europe	(Oral Health) AND (Disease OR Lesion) AND (Europe)	6,977	∼ 239	1,950	∼ 390
America	(Oral Health) AND (Disease OR Lesion) AND (America):	8,481	∼288	2,437	∼487
Asia	(Oral Health) AND (Disease OR Lesion) AND (Asia)	8,397	∼253	3,086	∼617
Africa	(Oral Health) AND (Disease OR Lesion) AND (Africa):	4,377	∼120	1,858	∼372

Based on these search results, out of the studies conducted in Europe over the past 20 years, 573 were clinical trials, 1,029 were cross-sectional studies, 3,997 were epidemiological studies, and 118 were systematic reviews. The search for studies in Asia included 643 clinical trials, 1,763 cross-sectional studies, 5,079 epidemiological studies, and 135 systematic reviews. The search for studies in the Americas included 570 clinical trials, 1,265 cross-sectional studies, 4,643 epidemiological studies, and 161 systematic reviews. The search for studies in Africa included 480 clinical trials, 692 cross-sectional studies, 2,385 epidemiological studies, and 126 systematic reviews (There was no review beyond 2006). The searches present a significant disparity in research output in Africa (about 37% less than Europe), which is a reflective indication of the 10/90 gap in oral health research within the continent, where less than 10 per cent of research funding addresses a challenge that affects a significant portion of the world population.

There's little to no information on oral health policies. However, a study by Gaffar et al. (2024) to examine their oral health policies reported that globally, 146 countries did not have policy documents on oral health (29). In Africa, only 10 countries have taken a position or have adopted an oral health policy, and according to a study by Gallagher et al. (2023), oral health professionals, including technicians, constituted only 1.11% of the health workforce within the continent, with a ratio of 0.44 professionals per 10,000 people (14).

Consequently, only a few Africans receive treatment for oral health problems, and with reduced annual visits by adults (20%–30%), oral health clinics are affected. The few studies of oral health visits in Africa should show low patronage and use of oral health services, and those who visit based on symptomatic reasons (23, 30). A survey conducted in urban Burkina Faso highlighted that 37.0% of adults had consulted an oral health clinic mainly for tooth removal (31), and a study in Nigeria asserted that 91% of Nigerian households do not use dental services (32).

## A neglected public health burden?

Oral health problems are becoming increasingly important in Africa. Over the last 30 years, Africa's population has grown by 49% (33). In the same manner, disease incidence has exponentially increased by 257 million in the last 30 years, with infants, young children, marginalised groups and the elderly being the most affected groups, leading to an ever-increasing oral disease burden, especially oral diseases such as gum diseases, dental cavities, and tooth loss. This is a reflection of unequal access to health services in the region (9). For example, the prevalence of Noma, a debilitating oral health disease of young children that affects the mouth and face, is found in extreme poverty regions in sub-Saharan Africa. When left untreated, it becomes very fatal. Despite the fact that most oral health diseases can be treated and prevented with healthy lifestyles, they continue to pose a significant health burden on vulnerable populations. This preventable health challenge leads to a negative aftermath affecting vulnerable groups through their inability to remain active in society, emotional trauma and loss of productivity. Thus, highlighting a potential failure of healthcare systems in protecting individuals at risk of oral diseases (34).

This failure can be associated with various institutional flaws in the African healthcare system, such as inadequate funding of public dental health institutions. According to WHO (2025), oral health is not regarded as a priority on the continent, and there is inadequate financial investment in it. In addition to this, Folayan et al. (2025) associated the lack of resource allocation to oral health in the African continent with the lack of policy and the low priority of oral health in general public health policy (26). This inadequate resource allocation to this sector hampers the ability to provide adequate patient care, as well as a chronic shortage of skilled manpower and a massive brain drain within the sector. Aside from this funding from governmental institutions, there seems to be a fundamental insurance coverage for oral health within the continent. According to a study by Agbor et al. (2024) in Yaoundé, crucial dental treatment such as dental prosthesis, orthodontics, etc, is not covered by insurance companies, and many insured individuals find it difficult to be reimbursed due to administrative bottlenecks (35). This raises the inability to afford the costs associated with proper oral healthcare, limiting accessibility to treatment options.

According to Solanki et al. (2019), the African population is expected to double or even triple with increased financial pressure on the vulnerable (old and young) who are trying to earn a living in a competitive market (36). This will reduce an even lesser capability to engage in oral checkups, as many individuals who experience oral health issues see it as a waste of scarce resources to visit professionals, and some will turn to self-medication for every toothache as a means of managing scarce resources (37). Therefore, there is a need to pay attention to oral health by African governments, researchers, and indigenous communities. In 2022, the World Health Assembly adopted a resolution on oral health committed to Universal health coverage for oral health, in which they pledged, among other global strategies, to develop ambitious national responses to promote oral health by 80% in 2030 and to reduce oral diseases, conditions and oral health inequalities by 10% (38). They attempted to do this by improving political commitment to oral health and allowing equal access to oral health by addressing social and commercial determinants and risk factors of oral diseases. The 3rd SDG goal of the Sustainable Development Goals, which involves a commitment to improve good health and well-being, also encompasses oral health (39). However, three years down the line, only 57% of African countries have abided by this policy and have set implementation strategies and targets, with Northern African countries taking the lead (26). Reports have shown that only a small fraction of national health budgets is allocated to oral health care. In addressing this gap, more attention needs to be paid to the needs of the oral health workforce, which are backed up by the development of oral health policies.

Researchers have an important role to play in helping direct research for context-specific evidence and guiding practice and policy towards oral health in Africa. This is especially true in sub-Saharan Africa, where there is a lacuna of longitudinal studies, clinical trials, and randomised controlled trials. This gap specifically pertains to studies that assess the effectiveness and cost-effectiveness of therapeutic interventions and oral epidemiology devoted to the oral health of the African people. A centralised focus on these aspects of oral health will assist policymakers on the continent in understanding the role of policy and health systems in responding to oral health challenges. They will also assess prevalence and risk factors of various oral health disorders among various groups, ranging from children to adults, vulnerable groups like individuals on the spectrum and individuals with physical and mental challenges. Furthermore, a collaborative effort is required to centralise African public health research. African public health journals need to add oral health to their priorities, e.g., calling for a series on primary oral health studies, and international journals can also assist with increased focus on African studies. While other international journals like BMC Oral Health have provided a platform for oral health, a more regionally targeted approach is crucial. This could be achieved by dedicating a series on primary oral healthcare, which streamlines research and challenges regionally, thus attracting funding to the field.

## Policy and research recommendations

### Policy priorities

- **Increase Funding Allocation** - African governments must urgently re-prioritise and significantly increase financial investment in oral healthcare, moving beyond the current fraction of national health budgets, which currently reflects a failure in protecting individuals at risk. This includes ensuring adequate funding for public dental health institutions and exploring mechanisms to improve fundamental insurance coverage for crucial dental treatments, which are currently often difficult to access or reimburse (9, 10).
- **Integrate Oral Health into Primary Health Care (PHC)** - oral health services, including prevention and early treatment for prevalent diseases like dental caries and periodontal disease, should be integrated seamlessly into broader PHC systems. This is crucial to improve access, particularly in rural and underserved areas, where populations are most vulnerable (12). Policy implementation should align with the WHO's 2022 resolution and the Sustainable Development Goal 3, focusing on achieving Universal Health Coverage for oral health (13)
- **Strengthen Workforce Training and Capacity** - policies must address the significant shortage and uneven distribution of the oral health workforce (currently 0.44 professionals per 10,000 people) through increased investment in training, retention, and managing the brain drain within the sector. Efforts should focus on developing the capacity of dental assistants and technicians, whose availability currently varies considerably across regions (14).

### Research priorities

- **National Surveys and Surveillance Systems** - invest in and implement robust oral health surveillance systems to address the current limitations in collecting robust incidence and prevalence data across many African countries. Prioritise the execution of national surveys to establish context-specific evidence and assess the prevalence and risk factors of various oral health disorders among diverse groups, from children to adults and vulnerable populations (17).
- **Longitudinal and Intervention Studies** - urgently address the lacuna of longitudinal studies, clinical trials, and randomised controlled trials (RCTs) within Africa and Low- and Middle-Income Countries (LMICs). Focus research on assessing the effectiveness and cost-effectiveness of therapeutic interventions for prevalent conditions like dental caries and periodontal disease, as well as oral epidemiology, to guide practice and policy.
- **Capacity Building for African-Led Research** - foster African-led research consortia to centralise public health research efforts and narrow the existing 10/90 research gap, which sees Africa with 37% less research output than Europe. African public health journals and international partners should dedicate journal sections or series on primary oral health studies to streamline research, address regional challenges, and attract necessary funding to the field (22).

## Conclusions

Africa bears one of the world's highest burdens of oral disease, yet accords it among the lowest public health priorities for its prevention and control. Profound knowledge gaps persist in basic epidemiology, risk factor profiles, intervention effectiveness, and health system responses, while policy gaps, absent financing, fragmented or non-existent national strategies, critical workforce shortages, and failure to integrate oral health into primary care continue to drive preventable suffering, disfigurement, and death, particularly among children, the rural poor, and marginalised communities. Closing these gaps demands immediate, evidence-based action: sustained investment in oral health financing, rapid workforce scale-up, full integration into primary health care, and robust national surveillance systems. African governments, regional bodies, donors, and researchers must collaborate to generate local evidence, implement proven interventions, and hold systems accountable for delivering essential oral health care as a human right. The 2022 World Health Assembly Resolution on oral health and Sustainable Development Goal 3 provides an unprecedented global mandate. Africa cannot afford to miss this window. Transforming oral health from a neglected burden into a marker of equitable progress is not only feasible, it is an ethical and public health imperative for the continent's future.

## Data Availability

The original contributions presented in the study are included in the article/Supplementary Material, further inquiries can be directed to the corresponding author.
